# Viscosity-Controllable Graphene Oxide Colloids Using Electrophoretically Deposited Graphene Oxide Sheets

**DOI:** 10.3390/mi13122157

**Published:** 2022-12-07

**Authors:** Jinseok Choi, Seong-Gyu Park, Yeo-Jin Choi, Seung-Mun Baek, Han-Jung Kim, Yoonkap Kim, Ki-Sik Im, Sung-Jin An

**Affiliations:** 1 Department of Advanced Materials Science and Engineering, Kumoh National Institute of Technology, Gumi 39177, Republic of Korea; 2 Nano Electronic Materials and Components Research Center, Gumi Electronics and Information Technology Research Institute, Gumi 39171, Republic of Korea; 3 Department of Green Semiconductor System, Daegu Campus, Korea Polytechnics, Daegu 41765, Republic of Korea

**Keywords:** graphene oxide, graphene oxide viscosity, electrophoretic deposition, viscosity increase, graphene oxide ink

## Abstract

Graphene oxide (GO) is one of the interesting ink materials owing to its fascinating properties, such as high dissolubility in water and high controllable electric properties. For versatile printing application, the viscosity of GO colloids should be controlled in order to meet the specific process requirements. Here, we report on the relatively rapid fabrication of viscosity-increased GO (VIGO) colloids mixed with electrophoretically deposited GO sheets (EPD-GO). As the GO colloid concentration, applied voltage, and deposition time increase, the viscosity of the GO colloids becomes high. The reason for the improved viscosity of GO colloids is because EPD-GO has parallel stacked GO sheets. The GO and VIGO colloids are compared and characterized using various chemical and structural analyzers. Consequently, our simple and fast method for the fabrication of GO colloids with enhanced viscosity can be used for producing inks for flexible and printed electronics.

## 1. Introduction

Graphene oxide (GO) as an oxide form of graphene is a cornerstone material among graphene-based materials owing to its superior dispersion in water [1]. GO, with its fascinating properties, can be applied to a variety of applications, such as refractive index sensors, plasmon resonance absorbers, electrochemical sensors, and adsorbent materials for environmental cleanup [2,3,4,5]. Although GO has electrically insulating properties due to its oxygen functional groups, its electrical conductivity can be easily restored by thermal or chemical treatments [6,7]. In this regard, over the past decade, GO colloids have been considered for printing electronics applications as potential inks. They have been used for producing flexible and printed electronics by adapting various printing techniques, such as ink jet printing, screen printing, gravure coating, and slot die coating [8,9,10,11,12,13,14]. Because these printing methods require different ink viscosities, the viscosity of the constituent GO solution is important. For example, inkjet printing, gravure printing, and screen printing require ink viscosities in the 10–20 mPa·s, 100–1000 mPa·s, and 500–5000 mPa·s ranges, respectively [9].

The viscosity of GO colloids is affected by the pH, sheet size, GO concentration, binder, and additives induced by modifying hydrogen bonding, π-stacking, and electrostatic interactions, as well as coordination [15]. Yeh et al. reported that the viscosity of GO solutions increased without any usage of binders [16]. Higher viscosity was observed for higher GO-weight solutions. First, a GO cake was produced using filtration. Subsequently, the GO cake pieces were dispersed in deionized (DI) water. The researchers obtained GO gels (>2 wt.%), doughs (>20 wt.%), and hard solids (>60 wt.%), using a continuum of GO–water mixtures with no added binders. Bai et al. studied the effects of pH, GO sheet size, and additives on the viscosity of GO solutions [15]. When the pH was close to 0.6, the viscosity increased dramatically owing to an increase in the electrostatic repulsion between the GO sheets. Furthermore, GO solutions with large lateral sizes (>2 μm) showed an increase in viscosity with increasing GO concentration. In contrast, there was no variation in the viscosity of the GO solutions with small lateral sizes (less than 1 μm). Most previous studies used high concentrations of GO (>2 wt.%) with added binder or another additive to produce highly viscous solutions [17,18,19]. The additives can improve the stability of the solutions and enhance the mechanical properties [20]. However, fast gelation is also an important requirement for hydrogel applications [21].

Herein, in this study, we report the process of mixing the deposited GO during the electrophoretic deposition (EPD) process with GO colloids, which is named electrophoretically deposited GO (EPD-GO), in order to increase the viscosity of the GO colloids. The viscosity of the colloids is enhanced according to the increased EPD-GO volume. Moreover, our process yielded a GO gel with 8 mg/mL. The characteristics of GO and viscosity-improved GO (VIGO) were analyzed using X-ray photoelectron spectroscopy (XPS), Raman spectroscopy, and Fourier transform infrared spectroscopy (FT-IR). Finally, the coating properties of two GO materials were compared using a metal mask.

## 2. Materials and Methods

### 2.1. Synthesis of GO

To synthesize GO, we used the well-known modified Hummers method [22]. First, 0.5 g of graphite flakes (graphite flake, Alfa Aesar, Haverhill, USA, ~325 mesh) were mixed with 20 mL of concentrated sulfuric acid (H_2_SO_4_, Daejung Chemicals, Siheung-si, Republic of Korea) in an Erlenmeyer flask. Then, 1.65 g of potassium permanganate (KMnO_4_, Daejung Chemicals, Siheung-si, Republic of Korea) was slowly added into these mixtures while stirring at 300 rpm for 10 min. The temperature of the mixture was maintained at 35 °C for 2 h. After stirring for 2 h, the temperature was let to drop below 10 °C, and 300 mL of DI water was added to the flask extremely slowly. Finally, hydrogen peroxide (H_2_O_2_, Daejung Chemicals, Siheung-si, Republic of Korea) was added to the flask until the solution turned yellow. The synthesized GO solution was washed with 10% hydrochloric acid and 50 mL of DI water. The filtered GO was dried in a convection oven at 60 °C for 1 day.

### 2.2. Fabrication of Viscosity-Increased GO Colloids

GO dispersions were prepared at concentrations in the 2–8 mg/mL range and then sonicated for 2 h to obtain GO colloids. In order to apply a direct current (DC) electric field to the GO colloids, 100-mesh stainless steel was used as the electrode materials (both anode and cathode), with a distance between the electrodes of 15 mm. The dimensions of the electrode in the experiment were 30 mm × 50 mm. A DC voltage in the 5–20 V range was applied to the GO colloids using a DC power supply; the voltage application time varied in the 1–30 min range.

### 2.3. Characterization

The viscosity of the GO colloids was measured using a viscometer with a cone-type spindle (Viscosmeter, Brookfield Ametek, Middleborough, USA, DVNext Cone and Plate Rheometer) at room temperature. The shear rate was 38.4 s^−1^, to compare the viscosity values of the GO colloids. The chemical and structural properties of the GO colloids before and after the viscosity change were investigated using Raman spectroscopy, XPS, and FT-IR. Finally, a simple pattern was formed on a polyethersulfone (PES) substrate by a simple doctor blade-coating method using a metal mask. After coating with GO and VIGO, the surface and thickness were compared and analyzed using a field emission scanning electron microscope (FE-SEM, FE-6500, JEOL, Tokyo, Japan).

## 3. Results

**Figure 1:**
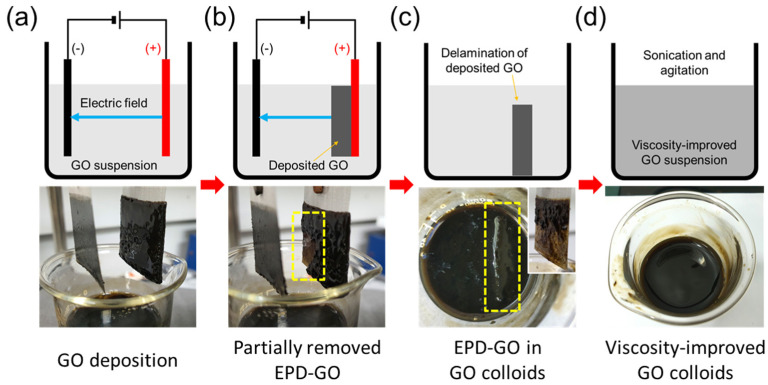
Schematic illustrations and photographs of the formation of viscosity-improved GO colloids. (**a**) Deposited GO on a SUS electrode. (**b**) Partially delaminated GO from an anode. (**c**) EPD-GO in a GO colloid. The inset shows the electrode after delamination of EPD-GO. (**d**) GO and EPD-GO mixed solution.

Figure 1 shows the schematic illustrations and photographs of the process that increased the viscosity of the GO colloids. GO sheets were deposited using an electric field on the anode, which is a well-known EPD method for GO sheets, as shown in Figure 1a. As the deposition of GO sheets proceeded, the deposited GO agglomerates were partially removed from the electrode; this is referred to as EPD-GO in the following section. Then, the EPD-GO were dropped into the GO colloids. The yellow dotted box in the photograph shown in Figure 1b indicates the partially removed GO sheets. Figure 1c shows the delaminated EPD-GO (yellow dotted box in the figure) in the colloids, as the over deposited GO sheets were removed from the electrode. The inset image of Figure 1c shows a thick delaminated GO layer on the anode.

**Figure 2:**
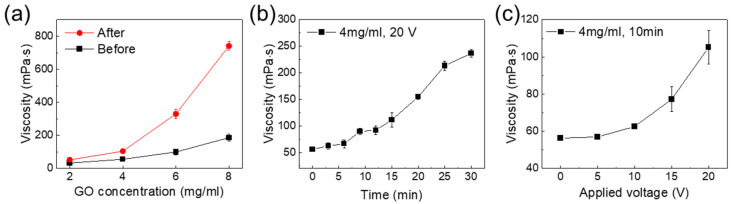
Viscosities of different GO colloids, as functions of the (**a**) concentration, (**b**) process time, and (**c**) applied voltage.

The viscosities of the GO colloids were measured in terms of the concentration, processing time, and applied voltage. Figure 2a shows the viscosities of the GO colloids before and after the EPD process, as a function of concentration. The applied voltage was 20 V and the process time was 10 min. Before the EPD process, the viscosities of the GO colloids with concentrations of 2, 4, 6, and 8 mg/mL were measured at 33, 56, 100, and 187 mPa·s, respectively. After the mixing with EPD-GO, these values increased to 52, 107, 325, and 763 mPa·s, respectively. The viscosity was higher for higher-concentration GO colloids. Figure 2b,c show the measured viscosities of the GO colloids according to the EPD process time and the applied voltage. The GO colloid viscosity increased with increasing process time and applied voltage. As the voltage application time increased, the measured viscosity gradually increased. The applied voltage was set to 5, 10, 15, and 20 V, the concentration of the GO colloid was set to 4 mg/mL, and the time was set to 10 min. The viscosities of the GO colloids were 57.11 mPa·s (5 V), 61.1 mPa·s (10 V), 79.11 mPa·s (15 V), and 107.4 mPa·s (20 V), while the viscosity was 56 mPa·s before the voltage application. These results indicate that the higher the applied voltage, the greater the viscosity change. As shown in Figure 1, the viscosity of the GO colloids increased with the addition of EPD-GO. The amount of the EPD-GO was related to the amount of GO deposited on the electrode during the EPD process. In previous studies it was found that the number of particles (w) deposited on the electrode during the EPD process can be expressed by the following Equation (1) [23]:

(1) $$w\;=\frac{2}{3}C\varepsilon_{0}\varepsilon_{r}\zeta \left(\frac{1}{\eta }\right)\left(\frac{E}{L}\right)t$$

where *C* is the concentration of the particles, *ε_0_* is the vacuum permeability, *ε_r_* is the permeability of the solution, *ζ* is the zeta potential of the particles, *η* is the viscosity, *E* is the applied voltage, *L* is the distance between electrodes, and *t* is the deposition time. The amount of deposition is mainly determined by the concentration of the solution, the applied voltage, and the deposition time. For this reason, as the concentration of the GO colloid increased, the applied voltage increased and the deposition time increased. Consequently, as the amount of EPG-GO increased, the GO colloid’s viscosity is also increased.

**Figure 3:**
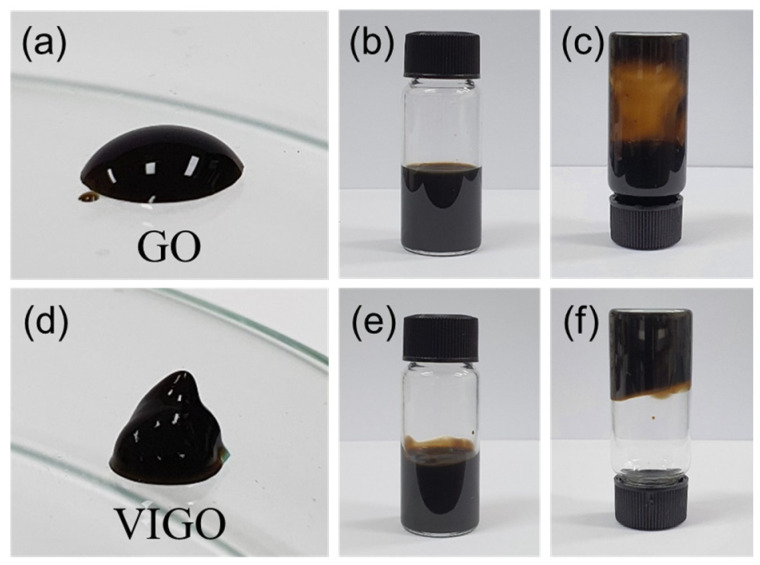
Photographs of dropped (**a**) GO and (**d**) VIGO droplets on a glass substrate. GO colloids in glass vials of GO with 8 mg/mL (**b**,**c**) and VIGO (**e**,**f**).

Figure 3 shows photographs of GO and VIGO at 8 mg/mL for 20 min. As shown in Figure 3a,d, there is a significant difference between the GO and VIGO droplet shapes on glass substrates. The GO droplets were hemispherical, as seen in general liquids. However, the VIGO droplets exhibited distinctly different shapes. The fluidity of VIGO was lost and the droplets were gel-like. In fact, even after 1 h, the VIGO droplets maintained the same shape, as the one shown in the figure. Figure 3b,c show GO in upright and upside-down vials, confirming that GO flowed straight down in the upside-down vial. On the other hand, VIGO did not flow down even in the upside-down vial, and remained at the same place, as shown in Figure 3e,f. In the GO solutions without additional binder, this phenomenon was observed for very high concentrations (approximately 30 mg/mL), but in this experiment, a nonflowing gel was obtained for concentrations as low as 8 mg/mL within 1 min [16]. Table 1 shows the comparison to the gel formation conditions in the literature.

**Figure 4:**
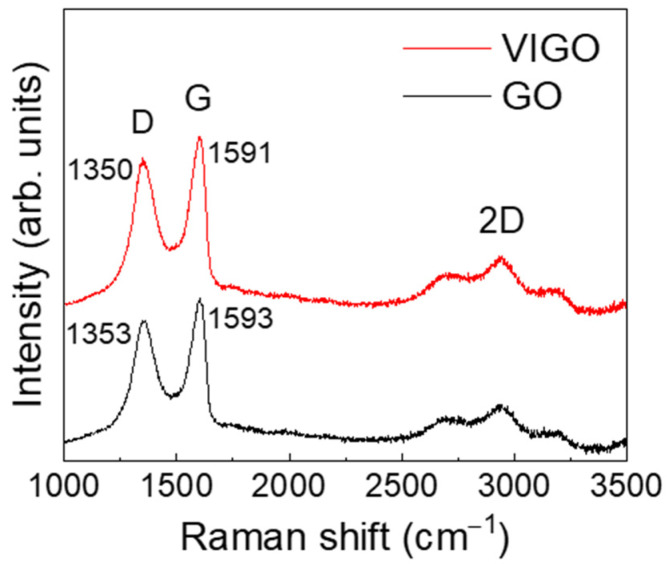
Raman spectra of GO and VIGO.

To clarify the increase in the viscosity, the chemical and structural properties of GO and VIGO were analyzed by Raman spectroscopy, XPS, and FT-IR. Figure 4 shows the Raman spectra of the GO and VIGO samples. The Raman spectrum of GO exhibited the D-band and G-band at 1353 cm^−1^ and 1593 cm^−1^, respectively. The D-band and G-band were attributed to the defects in graphene and sp^2^-hybridized carbon-based material, respectively [24]. The Raman spectrum of VIGO exhibited the D-band (1350 cm^−1^) and G-band (1591 cm^−1^). The D-band and G-band shifted toward lower wavenumbers for VIGO, compared with GO. This shift was observed for reduced GO obtained by electrochemical reduction [25]. Previously, our group identified a reduction in GO films using EPD. Because VIGO is a hybrid of GO and EPD-GO, the D-band and G-band shifted toward lower wavenumbers for VIGO compared with GO [26].

**Figure 5:**
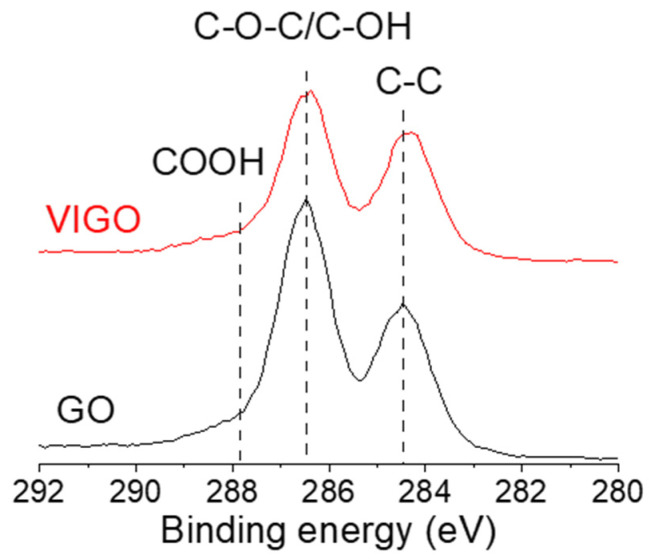
XPS C1s spectra of GO and VIGO.

Figure 5 shows the XPS C1s spectra of GO and VIGO. Both samples exhibited two dominant peaks at 284.4 eV and 286.7 eV, the peaks associated with sp^2^ C-C bond and epoxide (C–O–C)/hydroxyl (C–OH) groups, respectively [7]. In addition, a weak peak at 287.8 eV corresponded to carboxyl groups (–COOH). The XPS C1s spectra confirmed that the relative abundance of epoxide/hydroxyl groups was lower for VIGO compared with GO. In detail, we calculated the peak area to identify the ratio of carbon–carbon bonds (sp^2^ C-C bond) and carbon–oxygen bonds (epoxide/hydroxyl groups). The estimated ratio of the carbon–oxygen bonds was 67.4% for GO and 63.4% for VIGO. The peak corresponding to the carbon–oxygen bond of VIGO decreased to approximately 4% compared with GO. The lower abundance of carbon–oxygen bonds in VIGO could be explained as follows. The abundance of EPD-GO decreased during the EPD process, and our VIGO was a hybrid solution of GO and EPD-GO, constituting reduced GO [26]. Because of this, the relative abundance of carbon–oxygen bonds in VIGO was smaller compared to GO.

**Figure 6:**
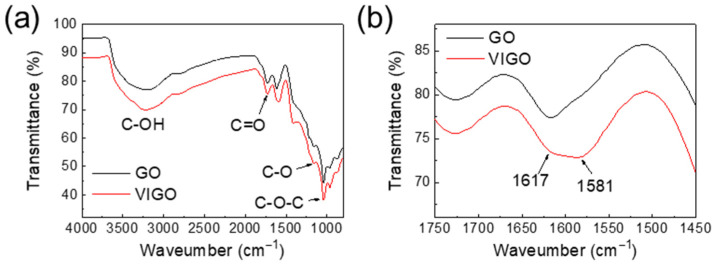
(**a**) FT-IR spectra of GO and VIGO. (**b**) FT-IR spectra of GO and VIGO, for wavelengths in the 1450–1750 cm^−1^ range.

Figure 6 shows the FTIR spectra of GO and VIGO. Figure 6a confirms the -OH bond at approximately 3220–3234 cm^−1^, C=O bonds at approximately 1723–1728 cm^−1^, C-O bonds at approximately 1412–1416 cm^−1^, and alkoxy bonds at approximately 1041 cm^−1^ [27]. Figure 6b shows the spectra in the 1450–1750 cm^−1^ range. Aromatic C=C bonds and adsorbed H_2_O were confirmed at 1581 cm^−1^ and 1617 cm^−1^, respectively [28,29]. In VIGO, stronger aromatic C=C absorption was observed than in GO. From the Raman and XPS analyses, VIGO exhibited a higher relative abundance of reduced GO than GO. Presumably, the partially reduced GO from EPD-GO caused stronger absorption of the aromatic C=C bond.

The viscosity change of GO colloids is caused by various factors, such as an increase in the GO concentration, acidification of the solution, large lateral size of the GO sheets, hydrogen bonding, and binding with polymeric substances or metal ions [15]. Bai et al. demonstrated that bonding interactions, such as hydrogen bonding, π-stacking, and electrostatic repulsion, affect the dispersion of GO sheets. In particular, a sufficiently high binding force can induce GO gelation. In our work, mixing EPD-GO increased the viscosity of GO colloids. When GO sheets were deposited on an electrode during the EPD process, the GO sheets formed frameworks through electrochemical reactions, and could become aligned [26]. For this reason, the viscosity of the GO colloids mixed with EPD-GO increased owing to the π-stacking of the GO sheets. Moreover, this phenomenon was confirmed for GO liquid crystals [30]. When the proportion of the nematic phase in GO colloids increased, the viscosity increased. Therefore, the oriented GO sheets of EPD-GO increased the viscosity of the GO colloids mixed with EPD-GO.

**Figure 7:**
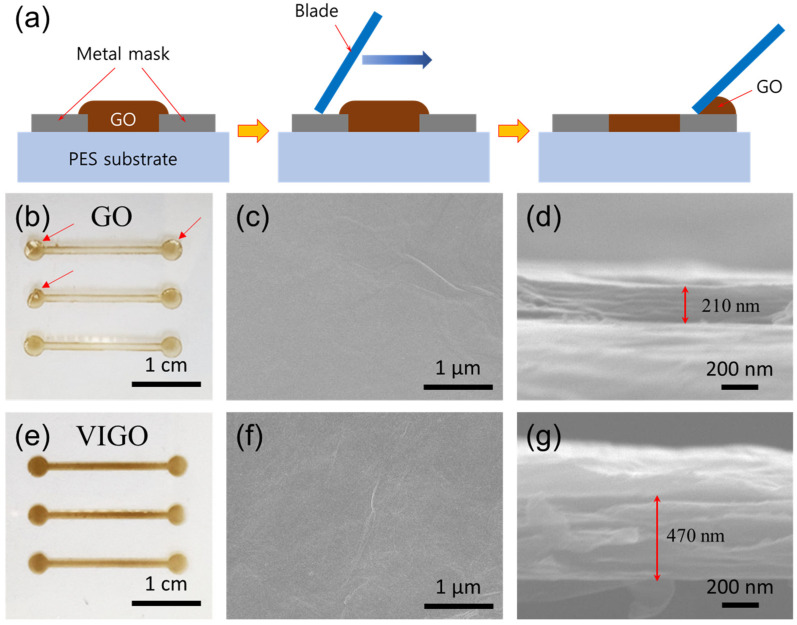
(**a**) Schematic of the GO and VIGO coating on PES substrates using a metal mask. (**b**) A photograph, (**c**) surface morphology, and (**d**) thickness of GO coated on a PES substrate. (**e**) A photograph, (**f**) surface morphology, and (**g**) thickness of VIGO coated on a PES substrate.

Finally, GO and VIGO were coated onto a PES substrate at 8 mg mL^−1^ using a shadow mask to form a simple pattern, as shown in Figure 7a. The coated GO and VIGO were then dried at 25 °C for 1 h. As shown in Figure 7b, some surfaces peeled off the substrate, which indicate red arrows. However, VIGO did not peel off the substrate at the edge, and it was confirmed that it was coated more clearly than GO. It was confirmed that there was no significant difference between the surfaces of the two samples. Figure 7d,g show the results of cross-sectional analysis, for each sample. The thickness of GO was approximately 210 nm (Figure 7b), whereas that of VIGO was 470 nm (Figure 7d). When the coating was performed using the same concentration of GO colloids, it was confirmed that the difference in thickness was at least twofold, depending on the difference in viscosity.

## 4. Conclusions

In conclusion, we demonstrated an increase in the viscosity of a GO colloid with a mixture of EPD-GO and GO with no additives. In our process, as the volume of EPD-GO increased, the viscosity of the colloid increased. Because the amount of EPD-GO is mainly determined by the concentration of the solution, the applied voltage, and the deposition time, as the concentration of the GO colloid increased, the applied voltage increased, and the deposition time increased, thus significantly increasing the viscosity of the GO colloid. Moreover, our process can be used for producing GO gels. When GO sheets were deposited on an electrode during the EPD process, the GO sheets formed frameworks through electrochemical reactions, and became aligned. Thus, EPD-GO exhibited a parallel stacked structure. Consequently, the oriented structure of EPD-GO increased the viscosity of the GO colloid. Our method offers a simple approach to viscosity improvement of GO colloids, allowing for their use as inks for flexible and printed electronics.

## Figures and Tables

**Table 1:** Comparison of various GO gel formation using additives.

Reference	Additives	GO Concentration	GO Form
[15]	Crosslinkers (PVP, HPC, PEO, etc.)	~5 mg/mL	Gel
[16]	- ^1^	Freeze-dried GO with 5 mg/mL	Gel, dough
[18]	Amino acid	5 mg/mL	Hydrogel
[19]	Sodium silicate	>60 mg/mL	Paste
This work	-	8 mg/mL	Gel

^1^ No additives.

## Data Availability

Not applicable.
